# Supplementary material for: Magnitude of Dural Tube Compression Still Does Not Show a Predictive Value for Symptomatic Lumbar Spinal Stenosis for Six-Year Follow-Up: A Longitudinal Observation Study in the Community
Source: J Clin Med. 2022 Jun 25;11(13):3668. doi: 10.3390/jcm11133668 (PMC9267800; doi:10.3390/jcm11133668)
Supplement: Supplementary file 1 [file jcm-11-03668-s001.zip › jcm-1648589-supplementary.pdf]

Table S1. Time course of LSS symptoms

			Six-year follow-up			Total
			Typical LSS-positive	LSS-negative		
				Insufficient LSS symptoms	No LSS symptoms	
Initial analysis	Typical LSS symptoms	Positive	13	18	21	52
		Insufficient	19	21	26	66
		No	28	40	46	114
Total			60	79	93	232

Abbreviations:

LSS: Lumbar Spinal Stenosis

Table S2. Relationship between change of typical LSS symptoms and the smallest DCSA

			The smallest DCSA (mm^2)				
Initial survey		Six-year follow-up	<25	25-49.9	50-74.9	75-99.9	100 ≤
Typical LSS symptoms	Positive	Positive	2	3	1	4	3
		Insufficient	8	4	0	4	4
		Negative	6	5	6	1	1
	Insufficient	Positive	1	5	5	3	5
		Insufficient	1	8	4	7	1
		Negative	2	9	3	5	7
	No	Positive	2	3	9	5	9
		Insufficient	1	5	11	8	15
		Negative	3	12	10	11	10
Total			26	54	49	48	55

Abbreviations:

DCSA: Dural sac Cross-Sectional Area   Av.: Average   CI: Confidence Interval   LSS: Lumbar Spinal Stenosis

Table S3. Relationship between change of LSS symptoms and the number of intervertebral discs whose DCSA was less than 50mm^2

			The number of intervertebral discs whose DCSA was less than 50mm^2			
Initial survey		Six-year follow-up	0	1	2	3≤
Typical LSS symptoms	Positive	Positive	8	2	3	0
		Insufficient	8	6	3	1
		Negative	8	5	4	4
	Insufficient	Positive	13	5	1	0
		Insufficient	12	5	3	1
		Negative	14	9	3	0
	No	Positive	23	3	1	1
		Insufficient	34	5	0	1
		Negative	31	9	4	2
Total			151	49	22	10

Abbreviations:

DCSA: Dural sac Cross-Sectional Area LSS: Lumbar Spinal Stenosis
